# Multivariate meta-analysis of mixed outcomes: a Bayesian approach

**DOI:** 10.1002/sim.5831

**Published:** 2013-04-30

**Authors:** Sylwia Bujkiewicz, John R Thompson, Alex J Sutton, Nicola J Cooper, Mark J Harrison, Deborah PM Symmons, Keith R Abrams

**Affiliations:** a Biostatistics Research Group, Department of Health Sciences, University of LeicesterUniversity Road, Leicester, LE1 7RH, U.K; b Genetic Epidemiology Group, Department of Health Sciences, University of LeicesterUniversity Road, Leicester, LE1 7RH, U.K; c Health Economics, Health Sciences - Methodology Research Group, School of Community Based Medicine, The University of ManchesterJean McFarlane Building, Manchester, M13 9PL, U.K; d NIHR Manchester Musculoskeletal Biomedical Research Unit, Arthritis Research UK Epidemiology Unit, School of Translational Medicine, University of ManchesterManchester, M13 9PT, U.K

**Keywords:** Bayesian analysis, multivariate meta-analysis, multiple outcomes, rheumatoid arthritis

## Abstract

Multivariate random effects meta-analysis (MRMA) is an appropriate way for synthesizing data from studies reporting multiple correlated outcomes. In a Bayesian framework, it has great potential for integrating evidence from a variety of sources. In this paper, we propose a Bayesian model for MRMA of mixed outcomes, which extends previously developed bivariate models to the trivariate case and also allows for combination of multiple outcomes that are both continuous and binary. We have constructed informative prior distributions for the correlations by using external evidence. Prior distributions for the within-study correlations were constructed by employing external individual patent data and using a double bootstrap method to obtain the correlations between mixed outcomes. The between-study model of MRMA was parameterized in the form of a product of a series of univariate conditional normal distributions. This allowed us to place explicit prior distributions on the between-study correlations, which were constructed using external summary data. Traditionally, independent ‘vague’ prior distributions are placed on all parameters of the model. In contrast to this approach, we constructed prior distributions for the between-study model parameters in a way that takes into account the inter-relationship between them. This is a flexible method that can be extended to incorporate mixed outcomes other than continuous and binary and beyond the trivariate case. We have applied this model to a motivating example in rheumatoid arthritis with the aim of incorporating all available evidence in the synthesis and potentially reducing uncertainty around the estimate of interest. © 2013 The Authors. Statistics inMedicine Published by John Wiley & Sons, Ltd.

## 1. Introduction

When the synthesis of data from studies reporting multiple outcomes is required, multivariate random effects meta-analysis (MRMA) can be used instead of performing meta-analyses on each outcome separately, which has an advantage of taking into account the correlations between the outcomes. Methods for MRMA have been developed for a number of purposes, for example, to estimate multiple outcomes from clinical trials 1, to model relationships between surrogate endpoints 2, or to evaluate diagnostic tests 3. Attention has mainly focussed on frequentist approaches, but methods, which use a Bayesian framework, have also been developed 2,4. In a recent review, Jackson *et al.* 5 describe advances in the development of the methodology of multivariate meta-analysis and discuss the advantages and disadvantages of the use of these methods. One of the advantages of integrating data on multiple outcomes through MRMA is that of ‘borrowing of strength’ across studies as well as across outcomes, which can potentially lead to reduced uncertainty around the resulting effectiveness estimates. In a Bayesian framework, a wider range of sources of evidence can be integrated. For example, additional data (external data from observational studies, clinical trials, or systematic reviews) or experts’ opinions can be incorporated in the form of prior distributions, which can further inform a multivariate meta-analysis model. The use of external data can potentially lead to a further reduction of uncertainty around the estimates. The main advantage of Bayesian analysis, however, is that it enables an analyst to combine evidence from multiple sources, which is an important factor in evidence-based medicine 6–8.

The MRMA models typically have a hierarchical structure in which the correlated multiple outcomes estimate underlying true effects (within-study model) and the multiple true effects are correlated and follow the same common distribution (between-study model). The elements of the within-study covariance matrices are assumed to be known; however, in practice, often only the variances are available. The correlations between the estimates (due to sampling variability), however, are rarely reported. The within-study correlations could be obtained if individual patient data (IPD) for all of the studies in MRMA were available, but this is hardly ever the case, and therefore, estimation of the within-study correlation is one of the major challenges in multivariate meta-analysis. Previous studies, which addressed this issue, included, for example, those suggesting the use of the average correlation obtained from the subset of those studies, which listed IPD 2, implementation of an alternative formulation of bivariate meta-analysis for studies with unknown within-study correlation, which combined covariances from both the within-study and the between-study model in a single term 9, or recently, a method of approximating the within-study covariances, based on the bivariate delta method, was developed 10. In contrast to the within-study covariance, the between-study covariance matrix is estimated from the model using study level data 11. However, in a Bayesian framework, the between-study covariance matrix or its elements require prior distributions to be placed on them (as do all the parameters: the between-study variances and correlations as well as the pooled effects).

Some of the previously developed Bayesian MRMA models were restricted to the bivariate case 2,12,13. Nam *et al.* 4 developed more general MRMA models; however, the examples used were also restricted to two outcomes. Arends *et al.* 14 proposed extension to trivariate meta-analysis, but the focus was mostly on a frequentist approach, and the use of Bayesian methods was only briefly discussed. In this article, we introduce a novel approach to MRMA in a Bayesian framework, which can be implemented for an extended number of outcomes and is presented here for a trivariate random-effects meta-analysis (TRMA) of mixed (continuous and binary) outcomes. This model allows us to incorporate data from a variety of sources, by not only including in the TRMA studies that are a mixture of those reporting all or only some of the outcomes but also by incorporating external data in the form of prior distributions. The use of informative prior distributions also extends previously developed methods, which used noninformative prior distributions, such as uniform distributions placed on correlations and inverse gamma distributions placed on variances, or Wishart prior distributions for precision matrices in models by Nam *et al.* 4. By contrast, we use external sources of data to construct informative prior distributions for both the within-study and between-study correlations. In our model, we assume that none of the studies in TRMA report the within-study correlations. We employ external IPD to construct prior distributions for the within-study correlations. To obtain the distributions of the correlations between mixed outcomes from the IPD, we used a double bootstrap method 15. We also describe methods of parameterization of the between-study model, which with an appropriate choice of prior distributions, allow the explicit incorporation of external information into the model through the Bayesian framework. Independent prior distributions are typically placed on the parameters of the multivariate model 2, but perhaps more appealingly, they can be placed on the correlations and other parameters in such a way as to account for their interdependence.

We apply this meta-analytical framework to an example from rheumatoid arthritis (RA). We carried out a meta-analysis of tumor necrosis factor-alpha inhibitors used sequentially in patients treated with RA. In studies assessing long-term effectiveness and/or cost-effectiveness of treatments in RA, the Health Assessment Questionnaire (HAQ) score, a self-reported measure of physical function, is of main interest as it is commonly used to estimate quality of life of patients following the treatment 16. In a systematic review, Lloyd *et al*. 17 found that very few studies reported the HAQ score, but there were other instruments used to assess patients’ responses to treatment. In the multivariate evidence synthesis models, we take into account data from studies reporting the HAQ score, as well as studies reporting other measures of response to treatment with the aim of integrating all available evidence and potentially decreasing uncertainty around the HAQ. We have devised a Bayesian framework in which external IPD is used to construct prior distributions for the within-study correlations between the HAQ and the alternative outcomes whereas external summary data (ESD) from a systematic review allows us to derive prior distributions for the between-study correlations.

The outline of this article is as follows. In Section 2, we introduce the motivating example and describe the data and the logic behind the meta-analysis model. Section 3 contains the details of trivariate meta-analysis model, including methods of constructing the prior distributions for the correlations. In Section 4, we briefly describe the implementation of these models in winbugs 18. We include the results of applying the model to the RA example in Section 5, which is followed by the discussion in Section 6. We describe further technical details, including the description of the multivariate model for any number of outcomes, in the Appendices.

## 2. Motivating example

### 2.1. Systematic review: ‘Lloyd data’

A systematic review and meta-analysis was carried out 17 to investigate the effectiveness of tumor necrosis factor-alpha inhibitors such as etanercept, infliximab, and adalimumab used as second line treatment in patients with RA. Standard instruments for measuring response to treatment in RA were considered: the HAQ and Disease Activity Score (DAS-28) measures, and the American College of Rheumatology (ACR) response criteria. The results of the meta-analyses of all three outcomes individually showed that the biologic interventions are effective when used sequentially. Data collected in this systematic review are used to investigate how multivariate meta-analysis can be applied to incorporate multiple outcomes, such as 20% response according to the ACR criteria (ACR20; a binary outcome used on log odds scale in MRMA) and changes from baseline of DAS-28 and HAQ scores (continuous outcomes), in evidence synthesis, which aims to estimate the change from baseline of the HAQ score. The estimate of the HAQ is of a particular interest to clinicians and decision-makers in health care as it is often used to estimate quality of life of patients following treatments of RA. Table 1 gives details of the three outcomes, which were reported in each of the studies within the systematic review. As indicated in the footnote of the table, standard errors (and occasionally the mean values) were often obtained from other measures or imputed. These unreported values could be treated as unknown in a Bayesian analysis. However, for the simplicity and exposition of this model, we chose to treat them as constant. The details of how these values were obtained can be found in Appendix 2 of the original meta-analysis of these data 17. We will refer to these data as the Lloyd data throughout this paper.

**Table I:** Studies in ‘Lloyd data’ reporting outcomes: 20% response according to the American College of Rheumatology criteria, Disease Activity Score, and Health Assessment Questionnaire.

Study	ACR20 r/n	DAS-28 Mean * (se)	HAQ Mean * (se)
Bennet 2005	—	− 1.7 (0.25)‡	− 0.31 (0.13)‡
Bingham 2009	85/188	− 1.6 (0.1)‡	− 0.35† (0.05)‡
Bombardieri 2007	486/810	− 1.9 (0.05)	− 0.48 (0.02)
Buch 2005	18/25	—	—
Buch 2007	55/72	− 1.47 (0.18)‡	—
Cohen 2005	—	− 1.87† (0.24)‡	—
Di Poi 2007	—	− 2.1† (0.29)‡	—
Finckh 2007	—	− 0.98 (0.18)‡	—
Haroui 2004	14/22	—	− 0.45† (0.14)‡
Hjardem 2007	—	− 1 (0.11)‡	—
Hyrich 2008	—	—	− 0.12 (0.03)‡
Iannone 2009	—	—	0.15† (0.13)‡
Karlsson 2008	172/337	—	—
Laas (InTol) 2008	—	− 1.17† (0.66)‡	—
Laas (InEff) 2008	—	− 1.26† (0.35)‡	—
Navarro-Sarabia 2009	—	− 1.1 (0.18)‡	− 0.21 (0.07)‡
Nikas 2006	18/24	− 2.4† (0.16)‡	—
Van der Bijl 2008	19/41	− 1.5 (0.25)	− 0.21 (0.08)
Van Vollenhoven 2003	12/18	—	—
Wick (EA) 2005	7/9	− 1.9 (0.22)‡	—
Wick (IA) 2005	19/27	− 1.3 (0.28)‡	—

ACR20, 20% response according to the American College of Rheumatology criteria; DAS-28, Disease Activity Score; HAQ, Health Assessment Questionnaire; se, standard error.

* mean change from baseline;

† mean values were obtained from other measures;

‡ standard errors were obtained from other measures or imputed as described in Appendix 2 of 17.

### 2.2. External individual patient data

None of the studies included in the Lloyd data reported correlations between outcomes, whereas such study-level correlations are required to fully specify a model of correlated outcomes. These correlations cannot be obtained directly from summary data, such as the Lloyd data, and none of the studies listed the IPD; thus, external data were required to estimate the correlations.

The IPD was obtained from the British Rheumatoid Outcome Study Group (BROSG) trial, which was designed to assess the benefit of aggressive disease-modifying antirheumatic drug treatments in patients with established RA, conducted by Symmons *et al.* 19. It was a randomized trial, which recruited 466 patients with stable RA. The trial assessed clinical outcomes (i.e., HAQ, DAS-28, and ACR20) in two cohorts of patients managed using either a regime focussed on symptomatic control of pain and stiffness in the shared care setting or a more aggressive regime focussing on control of symptoms and joint inflammation in the hospital setting.

The BROSG trial found no difference between the aggressive versus symptomatic treatment arms. Therefore, this data set may be used as a single cohort of 466 patients with established RA whose condition deteriorated modestly over a 3 year period. The data from the BROSG trial were used to construct prior distributions for the within-study correlations between outcomes in the multivariate models. Given the complexity of the models in this paper, only those 293 patients for whom all three clinical outcomes (HAQ, DAS-28, and ACR20) were reported were included in the analysis (to avoid further data imputation).

### 2.3. External summary data

In contrast to the within-study correlations, we can estimate the between-study correlations from the model using study level summary data. In a Bayesian framework, this could entail placing noninformative prior distributions on these correlations. However, one of the advantages of Bayesian approach we want to exploit here is the possibility of incorporating external information in the analysis. We can achieve this by using external information to construct informative prior distributions. To illustrate this, we conducted an *ad hoc* meta-analysis of ESD to obtain estimates of the between-study correlations, which can be used as prior distributions in our models (as described in Sections 3.4 and 3.5). The ESD included studies of the same type of treatment as in the Lloyd data but used as the first-line treatment. Supporting Information‡ include further details with the full list of studies included in the ESD.

### 2.4. Logic of the meta-analysis model and notation

**Figure 1:**
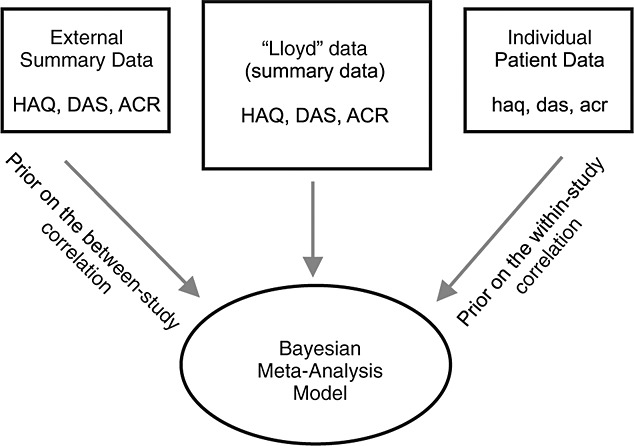
Structure of the data and the role of the data elements in the model.

In our motivating example, we aim to model the summary data of the correlated outcomes from the Lloyd data using a multivariate meta-analysis in a Bayesian form. To do so, we need to place prior distributions on the within-study and the between-study correlations (which are not known in the Lloyd data). We use the IPD, described in Section 2.2, to construct the prior distributions for the within-study correlations and the ESD, described in Section 2.3, to construct the prior distributions for the between-study correlations. Figure 1 illustrates this data structure and the role of each element within it. We use the external data to construct the prior distributions for the within-study and between-study correlations only. The remaining parameters of the model, such as the pooled effects and the between-study standard deviations, are given noninformative prior distributions 6. Note that the external data set used in this example was not very large. However, in more general circumstances, the relevance and rigor of the external evidence can be taken into account. For example, the variance of the prior distribution can be adjusted to construct a less informative distribution 6,20. In addition, when there are multiple external data sources, we can carry out a random effects meta-analysis. A number of authors have advocated using posterior predictive distribution from such external meta-analysis as a source of external evidence in the form of a prior distribution 6,21.

## 3. Trivariate random-effects meta-analysis

For the purpose of simplicity and direct link to the Lloyd data, the model presented here includes only three outcomes. The full multivariate model is described in Appendix A. Suppose that we have summary data available on at least one of three outcomes (*y*_1_, *y*_2_, and *Y*_3_). To be able to combine data from studies reporting these outcomes, we can carry out a meta-analysis of the three outcomes simultaneously. If we assume that all three outcomes are normally distributed (i.e., they are continuous outcomes or binomial outcomes on the log scale as for example log odds of response), we can model them simultaneously in the TRMA model (by extending bivariate models discussed by van Houwelingen *et al.* 22 and Riley *et al*. 23) as follows:


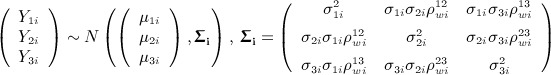
(1)


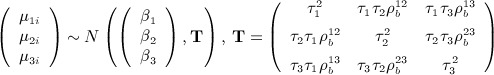
(2)

In the previous model, we assume outcomes *Y*_1*i*_, *Y*_2*i*_, and *Y*_3*i*_ to be estimates of correlated effects *μ*_1*i*_, *μ*_2*i*_, and *μ*_3*i*_ with corresponding within-study covariance matrices ***Σ***_**i**_ of the estimates. These study-level effects follow a trivariate normal distribution with means (*β*_1_,*β*_2_,*β*_3_) and covariance **T** in this hierarchical framework. We will refer to (1) as the within-study model and (2) as the between-study model. Prior distributions need to be specified for missing data (which we describe in Section 3.1), the within-study correlations (constructed in Section 3.2), and the elements of the between-study covariance matrix. The latter we propose to construct for an alternative parameterization of the between-study model as described in Section 3.3 with the choice of prior distributions discussed in Section 3.4 and finally constructed in Section 3.5.

Note that the bivariate random-effects meta-analysis (BRMA) is a special case of TRMA in (1)–(2), which can be obtained simply by reducing the dimension of TRMA, as described in Appendix B. If all the within-study correlations in TRMA, that is, the within-study correlations 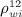, 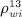, and 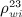, and the between-study correlations 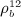, 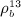, and 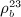 are equal to zero, then the model (1)–(2) reduces to three univariate random-effects meta-analysis (URMA) models for *Y*_1*i*_, *Y*_2*i*_, and *Y*_3*i*_:


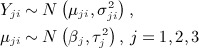
(3)

which in a Bayesian framework require specification of prior distributions such as the normal prior distribution for the mean effect *β*_*j*_ ∼ *N*(0,1000) and the half normal for the between-study standard deviation *τ*_*j*_ ∼ *N*(0.0,1000)*I*(0,) (where *N*( − , − )*I*( − ,) denotes the half normal distribution 6). Examples of alternative prior distributions for URMA can be found elsewhere 24.

### 3.1. Missing data

In our model, we assume that not all the studies report all the outcomes *Y*_1_, *Y*_2_, and *Y*_3_; that is, there are missing data for at least one of the outcomes for some of the studies. The advantage of conducting this meta-analysis in a Bayesian framework is that we give all the outcomes (whether known or missing) a distribution, which leads to these missing values being estimated directly from the model through the Markov Chain Monte Carlo (MCMC) simulation. Defining the trivariate model, as in (1), already provides distributions for missing values of *Y*_1*i*_, *Y*_2*i*_, and *Y*_3*i*_. However, corresponding missing standard deviations *σ*_1*i*_, *σ*_2*i*_, and *σ*_3*i*_ still need to be estimated. By assuming exchangeability of the variances, we can assume the corresponding population variances (rather than the variances of the mean) to come from the same distribution, for example,


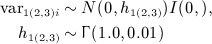
(4)

and 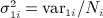, 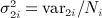, and 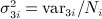. By ‘borrowing of information’ from the studies reporting the *Y*_1*i*_ (*Y*_2*i*_, *Y*_3*i*_) and their standard deviations, the variances (and hence standard deviations) for studies with missing *Y*_1*i*_ (*Y*_2*i*_, *Y*_3*i*_) are predicted by the MCMC simulation, conditional upon both the data and the posterior estimates of the model parameters.

### 3.2. Prior distributions for the within-study correlations

Clinical studies very rarely report the correlations between clinical outcomes, that is, the within-study correlations 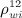, 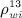, and 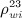; therefore, in the model (1), we assume them to be unknown. In the Bayesian framework, however, we must give the correlations prior distributions. These prior distributions can be either noninformative (such as a uniform distribution over range − 1 to 1) or more informative prior distributions potentially based on external information, for example, IPD containing observations on all outcomes from another, but relevant, study such as the IPD described in Section 2.2.

Suppose we have data available from an external study with individual participants for whom data on all three outcomes have been collected. We want to use IPD to construct prior distributions for the within-study correlations 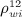, 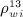, and 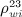. If all the outcomes in IPD were continuous, we could assume that they follow a common trivariate normal distribution and modeling them this way would allow us to evaluate the within-study correlation directly from the covariance matrix. Often, however, outcomes are a mixture of continuous ones and others, for example, binary ones (as is the case of ACR20, in our motivating example, which in the summary data is taken as log odds of ACR20 response, but in IPD, it is a binary outcome). Then, we cannot assume that the three variables follow a common trivariate normal distribution (unlike in the case of aggregate data in (1), we are unable to estimate log odds of response for individual patients).

There are two ways of overcoming this problem of constructing the prior distributions for the within-study correlations in TRMA. One is to use an approximation where the correlation between the mean of the continuous variable and the log odds of the binary one equals the correlation between the continuous observation and the binary observation. Another approach is to carry out a double bootstrap analysis. We have chosen the latter as it may be applied to other types of mixed outcomes. Double bootstrap methods have been developed to, for example, estimate confidence intervals 15,25. We have carried out a simulation, described in Appendix C, to show that this method can be applied to estimate summary statistics and the correlations between them.

We applied the double bootstrap method in the following way. From the IPD (containing observations on all three outcomes), we sampled *N*_*b*1_ = 500 bootstrap samples of size equal to that of the IPD, allowing for repetitions (first level bootstrap samples). From each of the bootstrap samples, we sampled another *N*_*b*2_ = 500 bootstrap samples (second level bootstrap samples). For each of the second level bootstrap samples, we calculated the mean values of the continuous variables and log odds of the binary ones. For the sets of summary statistics corresponding to each of the first level bootstrap samples, we then calculated the correlations between them. We used the simulated series of 500 sets of the three correlations (in the form of the 500 × 3 matrix) as a set of empirical prior distributions for the within-study correlations. In our main TRMA model, we sampled rows of this matrix (containing three correlations) to preserve the between-outcome correlation structure to ensure the nonsingularity of the within-study covariance matrices. In our example, the between-study variability exceeds the variability within the included studies; therefore, the estimates of the within-study correlations have little impact on the final results of TRMA 13, and hence, the same prior distributions for all within-study correlations in each of the studies could be used as in Nam et al 4 where the correlations do not vary with the study suffix *i*. However, in a more general scenario, this may not be appropriate. Therefore, for each of the studies in meta-analysis, we sample the three within-study correlations from different copies of the empirical prior distribution, thus allowing *ρ*_*wi*_ to have a different value for each study *i*, in every MCMC iteration.

Note that, as mentioned previously, if all three variables were continuous, it would be possible to estimate the within-study correlation directly (without the need for the bootstrap simulation). This is described for the bivariate version of the model in Appendix B.

### 3.3. Parameterization of the between-study model

For the between-study model (2), we need to specify prior distributions for the between-study variances 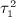, 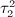, and 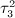, and the between-study correlations 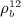, 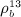, and 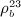. One approach, often adopted, is to give the inverse of the covariance matrix in (2), that is, the precision matrix **T**^ − 1^, a Wishart prior distribution 26. An alternative approach is to parameterize the between-study model (2) in the product normal formulation, that is, in the form of a product of a series of univariate conditional normal distributions 27. We chose this latter approach as it is more intuitive and allows us to use an explicit formulation of prior distributions for 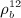, 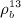, and 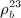 on the basis of external evidence. This approach is easier to implement, however, it requires making an additional assumption of independence of *y*_2_ and *Y*_3_ conditional on *y*_1_. This assumption means that elements (2,3) and (3,2) of the precision matrix are zero as the partial correlation coefficient between *y*_2_ and *Y*_3_ is zero 28. We express this formulation as follows


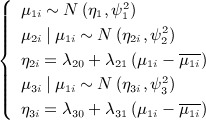
(5)

which can then be used to estimate the parameters of the between-study model (2):


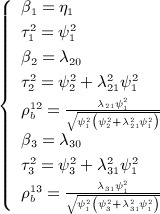
(6)

Note that the variable *μ*_1*i*_ in (5) has been centered to avoid high autocorrelation in the MCMC simulation.

### 3.4. Choice of the prior distributions for the between-study correlations

**Figure 2:**
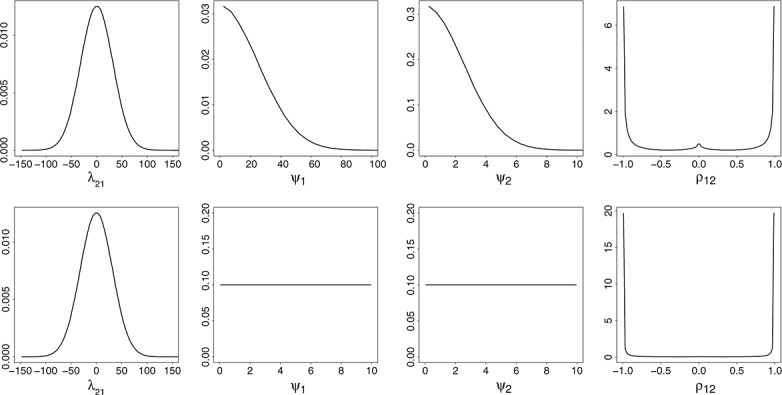
Examples of constructing prior distribution for the between-study correlation using independent noninformative prior distributions for the standard deviations and the regression coefficient, which can lead to implausible prior distribution for the correlation.

The formulae in (6) show the interdependencies between the parameters (i.e., the correlations, regression coefficients, and the standard deviations). Because they are inter-related, placing prior distributions on such parameters requires caution to ensure that they are plausible and realistic. For example, placing noninformative prior distributions on the standard deviations *ψ*_1_, *ψ*_2_, and *ψ*_3_ and the regression coefficients *λ*_20_, *λ*_21_, *λ*_30_, and *λ*_31_ in (5) may appear to be an appropriate approach; however, it can result in inadequately constructed prior distributions for the between-study correlations, 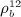, 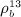 and 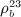, as shown in Figure 2 for the correlation between *y*_1_ and *y*_2_, 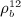. For example, using a normal prior distribution for the regression coefficient *λ*_21_ and an independent half normal distributions for the between-study standard deviations *ψ*_1_ and *ψ*_2_ can lead to a W-shaped prior distribution for the correlation (top row in Figure 2), whereas using a normal prior distribution for the regression coefficient and independent uniform distributions for the between-study standard deviations can lead to a U-shaped prior distribution for the correlation (bottom row in Figure 2). This choice of the hyper parameter for the prior distribution leads to sampling from extreme values of the correlation, also leading to poor convergence.

We found that in this case, it is more suitable to give the correlations an appropriate prior distribution first. Defining them together with the prior distributions for the between-study standard deviations *ψ*_1_, *ψ*_2_, and *ψ*_3_ enables evaluation of the regression coefficients *λ*_21_ and *λ*_31_ from (6):


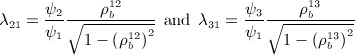
(7)

**Figure 3:**
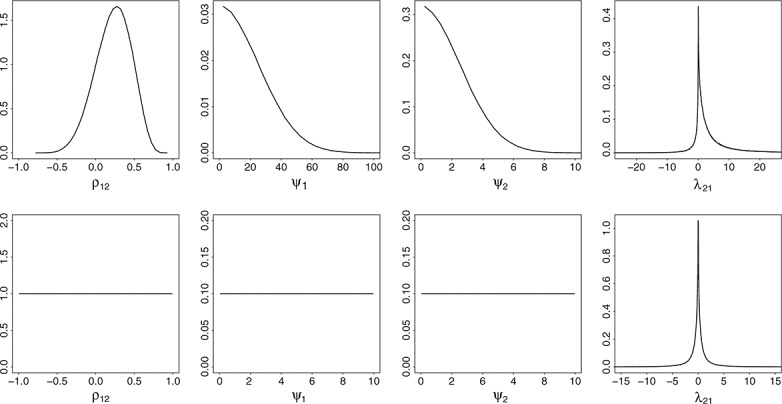
Examples of constructing prior distributions for the between-study model parameters using interdependent prior distributions for the parameters.

Figure 3 shows examples of such parameterizations of the prior distributions. Using an informative prior distribution for the between-study correlation (such as a normal prior distribution for the Fisher transformation of a correlation) and ‘vague’; for example, half normal distributions for the standard deviations *ψ*_1_ and *ψ*_2_ gives a more plausible implied prior distribution for the regression coefficient *λ*_21_ (top row in Figure 3). Another possible approach is to use the vague uniform prior distribution for the correlation as well as the between-study standard deviations, which also gives a plausible prior distribution for the regression coefficient (bottom row in Figure 3). Placing prior distributions first on 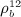 and 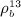 and on the between-study standard deviations *ψ*_1_, *ψ*_2_, and *ψ*_3_ and then estimating the implied prior distributions for the coefficients *λ*_21_ and *λ*_31_ largely improves the convergence of all the parameters, that is, the between-study correlations 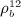 and 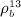 and the coefficients *λ*_21_ and *λ*_31_. We give the coefficients *λ*_20_ and *λ*_30_ noninformative prior distributions as they do not affect the correlations.

### 3.5. Construction of the prior distributions for the between-study correlations

We can use external data to obtain the prior distributions for the between-study correlations. We can obtain such data, for example, from a systematic review of studies (i.e., involving patients with the same condition and under comparable treatment) reporting all outcomes such as the ESD introduced in Section 2.3. A trivariate meta-analysis (1)-(2) is carried out on the three outcomes in ESD simultaneously in the same way as the main TRMA described in this section. Here, however, we choose to include only studies reporting all three outcomes; hence, there will be no missing data. We use vague prior distributions for the within-study and between-study correlations and the between-study standard deviations (obtaining informative prior distributions for these parameters would require another external data meta-analysis leading to an infinite process). We can use directly the posterior between-study correlations from this ESD analysis, 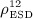 and 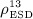, as the prior distributions for 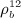 and 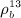 in (7). In the MCMC simulation of the main TRMA model, we sampled pairs of 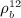 and 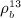 (thinned out to 500 pairs) to preserve the correlation structure between the conditionally dependent variables.

Having defined all prior distributions, we can obtain relevant estimates (posterior *β*_1_, *β*_2_, and *β*_3_ and the corresponding variances and correlations) from MCMC of the trivariate hierarchical model (defined in (1) and (2)).

## 4. Implementation in winbugs

We implemented all models in winbugs 18 where the estimates were obtained using MCMC simulation using 100,000 iterations (including 50,000 burn-in). We checked convergence by visually assessing the history, chains, and autocorrelation using graphical tools in winbugs and using the Geweke method in the BOA package 29. We present all posterior estimates as means with the 95% highest probability density intervals (HPDIs) except for the estimates displayed in the forest plots, which are means with the 95% credible intervals (CrI).

## 5. Results

We have applied the TRMA model to the Lloyd data to simultaneously model data on the HAQ, DAS-28, and ACR20 (with the IPD and ESD used to construct the prior distributions for the within-study and the between-study correlations between the outcomes, as described in Sections 3.2 and 3.5). In the Lloyd data, the HAQ and DAS-28 estimates represent the mean change from baseline of the outcomes, and ACR20 is a proportion of responders, which is transformed to log odds of response to be able to follow the normal distribution in TRMA. To investigate the impact of including more data in the analysis on uncertainty around the HAQ estimate, we explored results of the meta-analyses on three levels: using URMA of HAQ (as in (3)), bivariate random-effects meta-analysis (BRMA) combining the HAQ and the DAS-28 (as in (B1)-(B2) in Appendix B), and finally, TRMA by extending the data by ACR20. Table 2 lists the parameters for the prior within-study and between-study correlations used in both BRMA and TRMA. Table 3 shows the results obtained from all the models.

**Table II:** Prior correlations.

	Within-study correlations			Between-study correlations	
	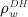	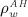	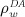	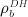	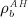
BRMA	0.24 [0.13,0.35]	—	—	0.86	—
				[0.46,0.999]	
TRMA	0.24 [0.10,0.38]	− 0.13	− 0.20	0.78	− 0.14
		[ − 0.29,0.0103]	[ − 0.31, − 0.08]	[0.27,0.998]	[ − 0.80,0.56]

BRMA, bivariate random-effects meta-analysis; TRMA, trivariate random-effects meta-analysis.

**Table III:** Results of the univariate meta-analyses of Health Assessment Questionnaire (HAQ), Disease Activity Score (DAS-28), and 20% response according to the American College of Rheumatology criteria (ACR20) separately, bivariate meta-analysis of HAQ and DAS-28, and trivariate of HAQ, DAS-28, and ACR20.

	Posterior mean (Standard error) [95% HPDI]				
	Univariate analyses				
	HAQ	DAS-28	ACR20	Bivariate HAQ & DAS-28	Trivariate HAQ, DAS-28, & ACR20
HAQ	− 0.25 (0.09)	—	—	− 0.28 (0.07)	− 0.28 (0.08)
	[ − 0.43, − 0.09]			[ − 0.41, − 0.14]	[ − 0.43, − 0.12]
					
DAS	—	− 1.57 (0.13)	—	− 1.51 (0.08)	− 1.52 (0.09)
		[ − 1.84, − 1.31]		[ − 1.67, − 1.35]	[ − 1.71, − 1.34]
					
*ACR*	—	—	0.62 (0.05)	—	0.61 (0.05)
			[0.53,0.71]		[0.52,0.71]
					
*τ*_*H*_	0.21 (0.09)	—	—	0.21 (0.07)	0.22 (0.09)
	[0.08,0.38]			[0.10,0.35]	[0.10,0.39]
					
*τ*_*D*_	—	0.44 (0.11)	—	0.44 (0.11)	0.44 (0.11)
		[0.25,0.67]		[0.24,0.67]	[0.25,0.67]
					
*τ*_*A*_	—	—	0.52 (0.19)	—	0.53 (0.19)
			[0.20,0.90]		[0.21,0.91]
					
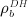	—	—	—	0.89 (0.12)	0.83 (0.18)
				[0.65,0.994]	[0.45,0.99]
					
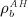	—	—	—	—	− 0.14 (0.30)
					[ − 0.63,0.49]

HPDI, highest probability density interval.

### 5.1. Results for bivariate random-effects meta-analysis of Health Assessment Questionnaire and Disease Activity Score

We carried out BRMA of HAQ and DAS-28 with the prior distributions for the within-study correlations estimated from the IPD (methods described in Appendix B) and for the between-study correlation estimated from the ESD (as in Section 3.5). The BRMA allowed us to include 10 cohorts (from the eight studies reporting DAS-28 but not HAQ in Table 1) in addition to those eight that could be used in URMA of HAQ (studies reporting HAQ in Table 1). The posterior correlation between DAS-28 and HAQ obtained from the IPD (used as a prior distribution for the within-study correlation in each study, as discussed in Section 2.2) was relatively weak, with mean 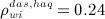 (95% HPDI [0.13,0.35]). The posterior correlation between DAS-28 and HAQ obtained from BRMA of the ESD (used here as a prior distribution for the between-study correlation) had a higher mean; 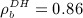 (95% HPDI [0.46,0.999]).

The HAQ estimate shifted toward a more extreme value when using BRMA (change in HAQ from baseline shifts from − 0.25 in URMA to − 0.28 in BRMA) with uncertainty reduced by 25% of the width of the 95% HPDI compared with the interval from URMA. The estimate of DAS-28, however, moved to a less extreme value when using BRMA (from − 1.57 to − 1.51), with reduced uncertainty. Adding more data to the meta-analysis by using BRMA reduced uncertainty but not heterogeneity; the between-study variances *τ*_*H*_ and *τ*_*D*_ remain almost the same, with only reduced uncertainty for *τ*_*H*_. However, the direct comparison of the variances with those from URMA is confounded by the different prior distributions being used: half normal prior distributions are used for *τ*_*H*_ and *τ*_*D*_ in URMAs, whereas in BRMA, half normal prior distributions are used for *ψ*_*H*_ and *ψ*_*D*_ (*ψ*_1_ and *ψ*_2_ in (5) for TRMA); hence, the implied probabilities for *τ*_*H*_ and *τ*_*D*_ are different.

**Figure 4:**
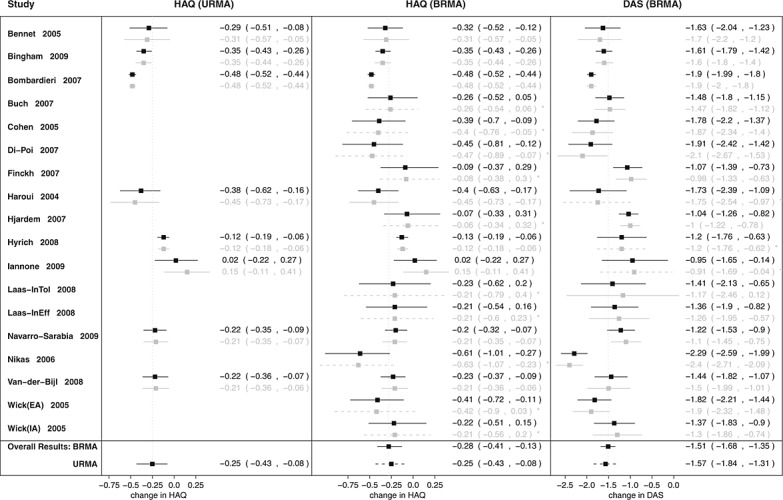
Forest plots for Health Assessment Questionnaire (HAQ): from univariate random-effects meta-analysis (URMA; left) and from bivariate random-effects meta-analysis (BRMA) of HAQ and Disease Activity Score (DAS-28; middle) and for DAS-28 also from BRMA (right). Graph shows estimates from the systematic review with 95% confidence intervals (grey solid lines), predicted missing estimates from BRMA with 95% credible intervals (CrIs; grey dashed lines), ‘shrunken’ estimates with 95% CrIs (black solid lines), and the pooled estimates with 95% CrIs (black solid lines for pooled effect from each of the meta-analyses and black dashed lines representing results from URMA for comparison).

**Figure 5:**
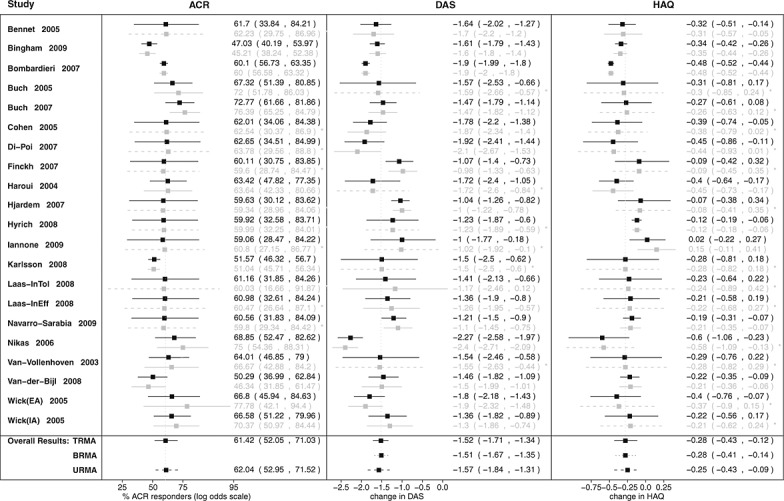
Forest plots for estimates of (from left to right) 20% response according to the American College of Rheumatology (ACR) criteria, Disease Activity Score (DAS-28), and Health Assessment Questionnaire (HAQ) from trivariate random-effects meta-analysis (TRMA) of HAQ, DAS-28, and ACR20. Graph shows estimates from the systematic review with 95% confidence intervals (grey solid lines), predicted missing estimates from TRMA with 95% credible intervals (CrIs; grey dashed lines), ‘shrunken’ estimates with 95% CrIs (black solid lines), and the pooled estimates with 95% CrIs (black solid lines for pooled effect from each of the TRMAs and black dotted (dashed) lines representing results from bivariate random-effects meta-analysis (univariate random-effects meta-analysis) for comparison).

Figure 4 shows three forest plots representing estimates of the HAQ from URMA (left) and BRMA (middle), and DAS-28 from BRMA (right). As in all forest plots (Figures 4 and 5), black solid lines correspond to the ‘shrunken’ estimates *μ*_*Hi*_ and pooled estimates *β*_*H*_, the grey solid lines show the estimates obtained from the systematic review (data used in this meta-analysis), and the grey dashed lines (estimates also marked with a * on their right) correspond to the predicted estimates for the studies that did not report the HAQ but reported the DAS-28 (or reverse in plots for the DAS-28). Dashed and dotted black lines below the pooled estimates represent the pooled estimates obtained from meta-analyses of reduced numbers of outcomes, for example, estimates from URMA below the estimates obtained from BRMA for comparison.

In the forest plot of the estimates from URMA, the estimates are shrunken toward the mean, which is especially noticeable for those studies with higher uncertainty around the known estimates (i.e., Bennet, Haroui, and Iannone). For example, in the case of the Haroui study, the HAQ estimate of − 0.45 (95% confidence interval (CI) [ − 0.73, − 0.17]) is now shifted toward the overall mean (which for URMA is − 0.26) with shrunken estimate equal − 0.38 (95% CrI [ − 0.63, − 0.15]). Borrowing of strength across studies leads to both the shift toward the mean and decrease in uncertainty reducing the width of the credible interval by 14% for the Haroui estimate. Note that the uncertainty corresponding to the pooled estimate is higher compared with the estimate obtained using the frequentist univariate meta-analysis 17; − 0.25 (95% CI [ − 0.4, − 0.11]). This is due to the between-study variance having a probability distribution (fixed in the frequentist approach) adding to the overall uncertainty in the model.

In BRMA, in addition to borrowing of strength across studies, there is also borrowing of strength between outcomes. In the middle forest plot in Figure 4 (representing estimates of HAQ from BRMA), the predicted estimates contribute to the pooled estimate even though there is considerable uncertainty associated with them. This additional borrowing of information across outcomes leads to the aforementioned reduction in uncertainty around the pooled estimate of the HAQ.

As can be seen in Figure 4 (middle and right-hand-side forest plots of estimates from BRMA), the predicted estimates of the HAQ (DAS-28) follow the heterogeneity pattern of the corresponding known estimates from the DAS-28 (HAQ) because of the informative (and of high mean) prior between-study correlation 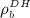 = 0.86 (95% HPDI [0.46,0.999]). Studies that reported the DAS-28, but not the HAQ, had on average relatively high positive estimates, which led to the high positive predicted estimates for the HAQ. Especially extreme values predicted for the HAQ were those for studies by Cohen, Di-Poi, Nikas, and one cohort of the Wick study, which had extreme values for the DAS-28. Also, two studies out of those three reporting the HAQ, but not the DAS-28, had more extreme estimates, showing little or no effect (only small improvement in HAQ in the Hyrich study and no improvement in Iannone), which led to the more extreme predicted estimates for the DAS-28 and, in consequence, reduced the pooled effect measured by the change from baseline of the DAS-28.

### 5.2. Results for trivariate random-effects meta-analysis of Health Assessment Questionnaire, Disease Activity Score, and 20% response according to the American College of Rheumatology criteria

We were able to include three further studies (which reported the ACR20, but not the HAQ or DAS-28 scores) by extending BRMA to TRMA. By incorporating an additional outcome, ACR20, we not only extended the data by those three studies but also incorporated the data on ACR20 from eight studies already in BRMA, which reported the HAQ and/or DAS-28 as well as ACR20 (the details can be found in Table 1). Adding ACR20 in TRMA did not, however, lead to any change in the point estimate of HAQ or its uncertainty; they remained almost the same as in BRMA. The between-study heterogeneity increased slightly (*τ*_*H*_ increased from 0.21 to 0.22) after inclusion of the three studies reporting the ACR20. This is likely due to the relatively high between-study heterogeneity of studies reporting the ACR20 and the prior correlation between the ACR20 and the HAQ being of rather low mean and relatively noninformative.

Figure 5 shows forest plots for all three outcomes: the ACR20, DAS-28, and HAQ. The predicted estimates (grey dashed lines) for the HAQ and DAS-28 follow the heterogeneity pattern of the corresponding known estimates of the DAS-28 and HAQ, respectively, as these are highly correlated outcomes, with the posterior between-study correlation 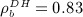 (95% HPDI [0.45,0.99]). However, the predicted estimates of the HAQ and DAS-28, for studies where neither of these outcomes were reported, are close to the overall mean with considerable associated uncertainty (as are the predicted estimates for ACR20 for studies reporting HAQ and/or DAS-28 but not ACR20). This is likely due to the lack of correlation between the ACR20 and the HAQ. This resulting level of uncertainty around the predicted estimates and the high between-study heterogeneity of studies reporting the ACR20 is the most likely explanation for the lack of a further reduction in uncertainty around HAQ when extending the analysis to TRMA. An additional forest plot showing all estimates of HAQ (obtained from all the models: URMA, BRMA, and TRMA) plotted together for comparison can be found in the Supporting Information.

## 6. Discussion

We have developed a Bayesian multivariate meta-analytic framework for incorporating data from multiple sources of evidence: studies reporting multiple mixed endpoints and additional external data, which are used to inform the parameters of the model in the form of prior distributions. The developed model gives a flexible way for combining data on mixed outcomes beyond the bivariate case. In this framework, a product normal formulation is adopted to describe the between-study variability of correlated outcomes. The advantage of this approach is the possibility of an explicit incorporation of external information to inform the prior distribution for the between-study correlation. However, as we have demonstrated, careful specification of the univariate prior distributions is essential so that the resultant prior distributions that are induced for the correlations are both plausible and realistic. We also show how individual patient data, consisting of mixed outcomes, can be used to construct empirical prior distributions for the within-study correlations. Our model comprised three parts as the prior distributions for the correlations resulted from the analyses of the two external data sets. It would be possible to carry out all components of the analysis within a single comprehensive Bayesian analysis. However, the flow of information would need to be restricted when putting the parts of the model together to prevent the main analysis from feeding back to, and influencing, the part of the model in which the empirical prior distributions were constructed 18. In our model, we used directly the posterior distributions of the relevant correlations, obtained from the analysis of external data, as the empirical prior distributions for the correlations. By sampling directly from the posterior distributions, we avoided the potential problem of having to make distributional assumptions to summarize the prior external evidence.

It has been previously discussed 30 that applying a multivariate approach to meta-analysis makes little difference to the results compared with those from the univariate approach. However, our findings show that this is clearly not always the case, and the multivariate approach can be of practical importance. We found that employing this Bayesian approach to evidence synthesis of outcomes in RA by combining the HAQ endpoint with the DAS-28 leads to a significant reduction in the uncertainty around the HAQ from − 0.25 (95% HPDI [ − 0.43, − 0.09]) obtained from URMA to − 0.28 (95% HPDI [ − 0.41, − 0.14]) obtained from BRMA. We have obtained similar results when using TRMA of the HAQ, DAS-28, and ACR20. The inclusion of the third outcome, ACR20, did not contribute to the HAQ estimate or reduction of uncertainty around it.

We cannot predict the extent of the gain in the precision of the pooled estimate, and the inclusion of additional outcomes through a multivariate approach does not always lead to reduced uncertainty around the estimate of the outcome of interest. In fact, in our example, extending the bivariate meta-analysis to the three-dimensional case did not contribute to a further reduction in uncertainty around the clinical outcomes. The additional number of studies included in the meta-analysis, by extending the analysis to multiple outcomes, can also lead to additional heterogeneity and therefore potentially *increase* the level of uncertainty. Sturtz *et al.* 31, have discussed a similar effect in the context of mixed treatment comparison meta-analysis where extending a network (by inclusion of additional interventions and studies) can sometimes lead to increased uncertainty around the estimates because of increased heterogeneity. However, there are other potential advantages of using a multivariate approach. It has been recently suggested by Kirkham *et al.* 32 that under some circumstances, a multivariate approach to meta-analysis can lead to a more appropriate estimate of the clinical outcome in the presence of outcome reporting bias. This may apply to the case of RA (discussed previously) where, because of the availability of a number of instruments for measuring the disease activity or response to treatment, authors may choose to report only those outcomes whose estimates are positive and/or significant. Although, in our model, we make no assumption regarding outcome reporting bias, this multivariate approach may reduce the effect of such bias if it existed.

One of the limitations of this particular example in RA is that the IPD comes from a study with treatments, which are very different from those used in the Lloyd data. It is possible that not only is the treatment difference an issue, but also that patients did not respond particularly well to the treatment and there was relatively little variability between patients leading to a low correlation. However, despite this limitation, we chose to use these prior distributions as they serve the purpose of illustrating how IPD can be used to construct a set of empirical prior distributions for the whole correlation structure of the within-study correlations. However, it is not always possible to obtain relevant individual patient data. Nevertheless, the Bayesian approach has another advantage as it allows elicitation techniques to be used to incorporate experts’ opinions in the form of prior distributions in a model 33. For example, methods for inclusion of expert opinion on bias in the synthesis of studies have been developed 20.

We can apply the modeling framework presented in this article to a range of settings in evidence-based medicine. For example, alternative outcomes such as surrogate endpoints are increasingly considered in decision-making in health care, especially in the early stages of the drug development process when extended follow-up time is required to obtain the main clinical outcome of interest. In addition to the multivariate framework as a way of combining data on multiple correlated outcomes, the Bayesian approach gives another level of integrating diverse sources of evidence, as it allows for incorporation of external information in the form of prior distribution. In our view, this approach is an important step toward the coherent synthesis of multiple sources of evidence, which is vital in policy decisions in health care 7.

## Appendix A. Multivariate random-effects meta-analysis

Suppose that we have summary data available on at least one of *N* outcomes (*y*_1_, *y*_2_, , *Y*_*N*_). If we assume that all outcomes are normally distributed (i.e., they are continuous outcomes or transformations of other outcomes), we can model them simultaneously in the MRMA model (by extending TRMA model described in Section 3) as follows:

(A1)

(A2)

In the this model, outcomes *Y*_1*i*_, *Y*_2*i*_, , *Y*_*Ni*_ are assumed to be estimates of correlated effects *μ*_1*i*_, *μ*_2*i*_, , *μ*_*Ni*_ with corresponding within-study covariance matrices ***Σ***_**i**_ of the estimates. These study-level effects follow a multivariate normal distribution with means  and covariance **T** in this hierarchical framework. Equations (A1) and (A2) describe the within-study and the between-study models, respectively.

### A.1. The between-study model in the product normal formulation

Similarly as for TRMA, by assuming independence of *y*_2_, , *Y*_*N*_ conditional on *y*_1_, we can parameterize the between-study model (A2) in the form of product normal formulation as follows:

(A3)

which can then be used to estimate the parameters of the between-study model (A2)

(A4)

Rearranging the formulas in (A4) leads to the following relationships between the regression coefficients, standard deviations, and the between-study correlations:

(A5)

which can be used to define inter-related prior distributions in a similar manner as described in Section 3.4 for TRMA.

## Appendix B. Bivariate random-effects meta-analysis

In the algebra in this section, we adopt the following notation. For the summary data, we denote the outcomes in the upper case: The variables *y*_1_ and *y*_2_ represent the mean values of the continuous outcomes. While when referring to the individual observations in Section B.1, we use the lower case: Variables *y*_1_ and *y*_2_ are observations for an individual.

We obtain the model for BRMA by reducing the dimension of TRMA in the following form:

(B1)

(B2)

In this model, similarly as in TRMA, we assume outcomes *y*_1_ and *y*_2_ to be estimates of correlated effects *μ*_1*i*_ and *μ*_2*i*_ with corresponding covariance matrices ***Σ***_**i**_. These study-level effects follow a bivariate normal distribution with means (*β*_1_,*β*_2_) and covariance **T**. We predict the missing outcomes and standard deviations in the same manner as for TRMA (as described in Section 4.1). We can obtain the prior distributions for the within-study and the between-study correlations in the same manner as for TRMA. However, when both outcomes *y*_1_ and *y*_2_ are continuous, we can obtain the within-study correlation from an analytical model described below.

### B.1. The within-study correlation

When both outcomes are continuous, we can obtain prior distribution for the within-study correlation by solving an analytical bivariate model for IPD, which will give a posterior distribution for the correlation between the patients’ outcomes. We can then use this posterior distribution directly as a prior distribution for the within-study correlation.

Suppose we have data available from an external study with  participants, for whom data on both outcomes have been collected. Because the outcomes are correlated, we can estimate both outcomes from a bivariate model, assuming that the two outcomes simultaneously follow a bivariate normal distribution (we use the lower case for *y*_1_ and *y*_2_ to distinguish the change from baseline for individuals in IPD from the mean values in aggregate data in meta-analysis, which have been denoted in the upper case):

(B3)

where *θ*_1_ and *θ*_2_ are mean values for *y*_1_ and *y*_2_ and Ω is the covariance matrix with the study-level correlation , whose estimate we will use to define prior distributions for the within-study correlations  in (B1).

We can parameterize the model (B3) in a product normal formulation as

(B4)

and prior distributions for this model are: *γ*_1_, *α*_0_, *α*_1_ ∼ *N*(0,0.001), *ξ*_1_,*ξ*_2_ ∼ *U*(0,10), which are meant to be relatively noninformative. Estimates from this parameterization can then be used to obtain the parameters of the model (B3) as follows:

(B5)

We can use the posterior correlation  obtained from (B5) directly (in the same model and code) as a prior distribution for the within-study correlations  in (B1) in a coherent way with no need to make further assumptions about the family of the distribution. Alternatively, we can obtain the prior correlation by calculating the Fisher transformation . We can assume the Fisher transformed correlation to be normally distributed; , and then, we obtain the prior distribution for the between-study correlation by back-transforming *ρ*^*FT*^ into ).

## Appendix C. Simulation to test the double bootstrap method for summary statistics

We have carried out a simulation to show that a double bootstrap method 15,25 can be applied to estimate correlation between summary statistics with uncertainty from individual subject data containing correlated observations. For the simulation, we chose to use the bivariate case of normally distributed data as in the normal case we know what the correlations between the summary statistics are: The correlation between the normally distributed individual observations equals the correlation between the means.

We simulated data, of size *N*, containing two correlated variables:

(C1)

For these data, we calculated the correlation *r* and corresponding confidence interval by transforming the correlation (using the Fisher transformation):

(C2)

We obtained the standard error using the formula: , and then, the confidence interval for the transformed correlation is

(C3)

This confidence interval is then back-transformed to obtain the confidence interval for the correlation:

(C4)

Using the simulated data, we then performed a double bootstrap simulation in the following way. From the simulated data, we sampled *N*_b1_ bootstrap samples. From each of the bootstrap samples, we sampled another *N*_b2_ bootstrap samples (second level bootstrap samples). For each of the second level bootstrap samples, we calculated the mean values of the outcomes. We calculated the correlations between the means: . We obtained confidence interval using the Fisher transformation in the same manner as for the correlation *r* previously. We then compared  with *r*.

We ran this simulation for the following example: *N* = 1000, *y*_1_ ∼ *N*(0,5), *y*_2_ ∼ *N*(2 + 4 * *y*_1_,10). We obtained the following results: *r* = 0.946, (95% CI: [0.939;0.952]) for the correlation obtained from the data directly and *ρ* = 0.946, (95% CI: [0.939;0.953]) for the correlation obtained from the double bootstrap simulation with *N*_*b*1 / *b*2_ = 5000 bootstrap samples on both levels.
