# Supplementary material for: Multivariate meta-analysis of mixed outcomes: a Bayesian approach
Source: Stat Med. 2013 Apr 30;32(22):3926–43. doi: 10.1002/sim.5831 (PMC4015389; doi:10.1002/sim.5831)
Supplement: Supplementary file 1 [file sim0032-3926-sd1.pdf]

Research Article

Statistics in Medicine

Received XXXX

(www.interscience.wiley.com) DOI: 10.1002/sim.0000

Supporting Web Materials to Multivariate meta-analysis of mixed outcomes: a Bayesian approach

Sylwia Bujkiewicz[a]* John R.Thompson[b], Alex J. Sutton[a], Nicola J. Cooper[a], Mark J. Harrison[c], Deborah P. M. Symmons[d] and Keith R. Abrams[a]

1. External summary data

A multivariate meta-analysis of external summary data (ESD) was carried out in order to construct the prior distributions for the between-study correlations between the outcomes in the rheumatoid arthritis (RA) study described in Section 2 of the Main Manuscript. The external studies included those from a technology appraisal of adalimumab, etanercept and infliximab in treatment of RA in biologically naive patients, carried out by Chen *et al.* [1], provided that suitable data on the HAQ and at least one of the alternative outcomes: DAS-28 or ACR20 were available. We extended the data from this review by including two additional cohorts. One study, which was excluded from the technology appraisal due to being an observational study, has been included in this analysis as the exclusion criteria did not apply here (it is a single-arm meta-analysis) and the study provided valuable data. Also, one of the studies from the "Lloyd data" (Bombardieri) containing a large control cohort of biologically naive patients reported the outcomes of interest; we included this cohort in ESD as only patients using the biologics as second line therapy have been included in the "Lloyd data". Table 1 lists the data collected to inform these prior distributions as described in Section 2.3 of the Main Manuscript.

¡[a]*Biostatistics Research Group, Department of Health Sciences, University of Leicester, University Road, Leicester, LE1 7RH, UK*
[b]*Genetic Epidemiology Group, Department of Health Sciences, University of Leicester, University Road, Leicester, LE1 7RH, UK* [c]*Health Economics, Health Sciences - Methodology Research Group, School of Community Based Medicine, The University of Manchester, Jean McFarlane Building, Manchester, M13 9PL, UK* [d]*NIHR Manchester Musculoskeletal Biomedical Research Unit, Arthritis Research UK Epidemiology Unit, School of Translational Medicine, University R1of Manchester, Manchester, M13 9PT, UK*
* *Correspondence to: Department of Health Sciences, University of Leicester, University Road, Leicester, LE1 7RH, UK. E-mail: sb309@le.ac.uk*

Contract/grant sponsor: This work was supported by the UK National Institute for Health Research [NF-SI-0508-10061 to K.R.A.] and the Medical Research Council [G0800770 to A.J.S, N.J.C. and K.R.A]. The BROSG trial was funded by the National Health Service (NHS) Executive, UK [NHS Technology Assessment project 94/45/02 to D.P.M.S.].

Copyright © 0000 John Wiley & Sons, Ltd.

Table 1. Studies reporting outcomes: ACR20, DAS-28 and HAQ

Study	ACR20<br>r/n	DAS-28<br>mean* (se)	HAQ<br>mean* (se)
Bombardieri 2007 [2]	3731 / 5711	-2.2 (0.018)	-0.55 (0.01)
Cohen 2004 [3]	12 / 18	-2.5 (0.17)	-0.6 (0.15)
Rau 2004 [4] i.v.	13 / 18	-0.82 (0.11)	-0.27 (0.15)
Rau 2004 [4] s.c.	12 / 18	-0.65 (0.18)	-0.1 (0.15)
van de Putte 2003 [5]	115 / 284	-2 (0.08)	-0.46 (0.03)
van de Putte 2004 [6] 20mg eow	38 / 106	-1.3 (0.16)	-0.29 (0.06)
van de Putte 2004 [6] 20mg qw	44 / 112	-1.6 (0.16)	-0.39 (0.06)
van de Putte 2004 [6] 40mg eow	52 / 113	-1.7 (0.15)	-0.38 (0.06)
van de Putte 2004 [6] 40mg qw	55 / 103	-2 (0.16)	-0.49 (0.05)

* mean change from baseline
i.v. = intravenous; s.c. = subcutaneous; qw = once weekly; eow = every other week

2. Additional results

Figure 1 shows combined results for HAQ (the main outcome of interest), which are the estimates from all of the models: univariate meta-analysis of the HAQ, bivariate of the HAQ and DAS-28 and trivariate of HAQ, DAS-28 and ACR20, which are discussed in the Main Manuscript.

References

1. Chen Y-F, Jobanputra P, Barton B, Jowett S, Bryan S, Clark W, Fry-Smith A, Burls A. A systematic review of the effectiveness of adalimumab, etanercept and infliximab for the treatment of rheumatoid arthritis in adults and an economic evaluation of their cost-effectiveness. *Health Technology Assessment* 2006; **10**:(42), i–250.
2. Bombardier S, Ruiz AA, Fardellone P, Geusens P, McKenna F, Unnebrink K, Oezer U, KaryS, Kupper H, Burmester GR Effectiveness of adalimumab for rheumatoid arthritis in patients with a history of TNF-antagonist therapy in clinical practice. *Rheumatology* 2007; **46**:1191–9.
3. Cohen JD, Zaltni S, Kaiser MJ, Bozonnat MC, Jorgensen C, Daures JP, Sany J Secondary addition of methotrexate to partial responders to etanercept alone is effective in severe rheumatoid arthritis. *Ann Rheum Dis* 2004; **63**:209-10.
4. Rau R, Simianer S, van Riel PL, van de Putte LB, Kruger K, Schattenkirchner M, Allaart CF, Breedveld FC, Kempeni J, Beck K, Kupper H Rapid alleviation of signs and symptoms of rheumatoid arthritis with intravenous or subcutaneous administration of adalimumab in combination with methotrexate. *Scand J Rheumatol* 2004; **33**:145-53.
5. van de Putte LB, Rau R, Breedveld FC, Kalden JR, Malaise MG, van Riel PL, Schattenkirchner M, Emery P, Burmester G, Zeidler H, Moutsopoulos H, Beck K, and Kupper H Efficacy and safety of the fully human anti-tumour necrosis factor alpha monoclonal antibody adalimumab (D2E7) in DMARD refractory patients with rheumatoid arthritis: a 12 week, phase II study. *Ann Rheum Dis* 2003; **62**:1168-77.
6. van de Putte LB, Atkins C, Malaise M, Sany J, Russell AS, van Riel PL, Settas L, Bijlsma J, Todesco S, Dougados M, Nash P, Emery P, Walter N, Kaul M, Fischkoff S, Kupper H Efficacy and safety of adalimumab as monotherapy in patients with rheumatoid arthritis for whom previous disease modifying antirheumatic drug treatment has failed. *Ann Rheum Dis* 2004; **63**:508-16.

Copyright © 0000 John Wiley & Sons, Ltd.

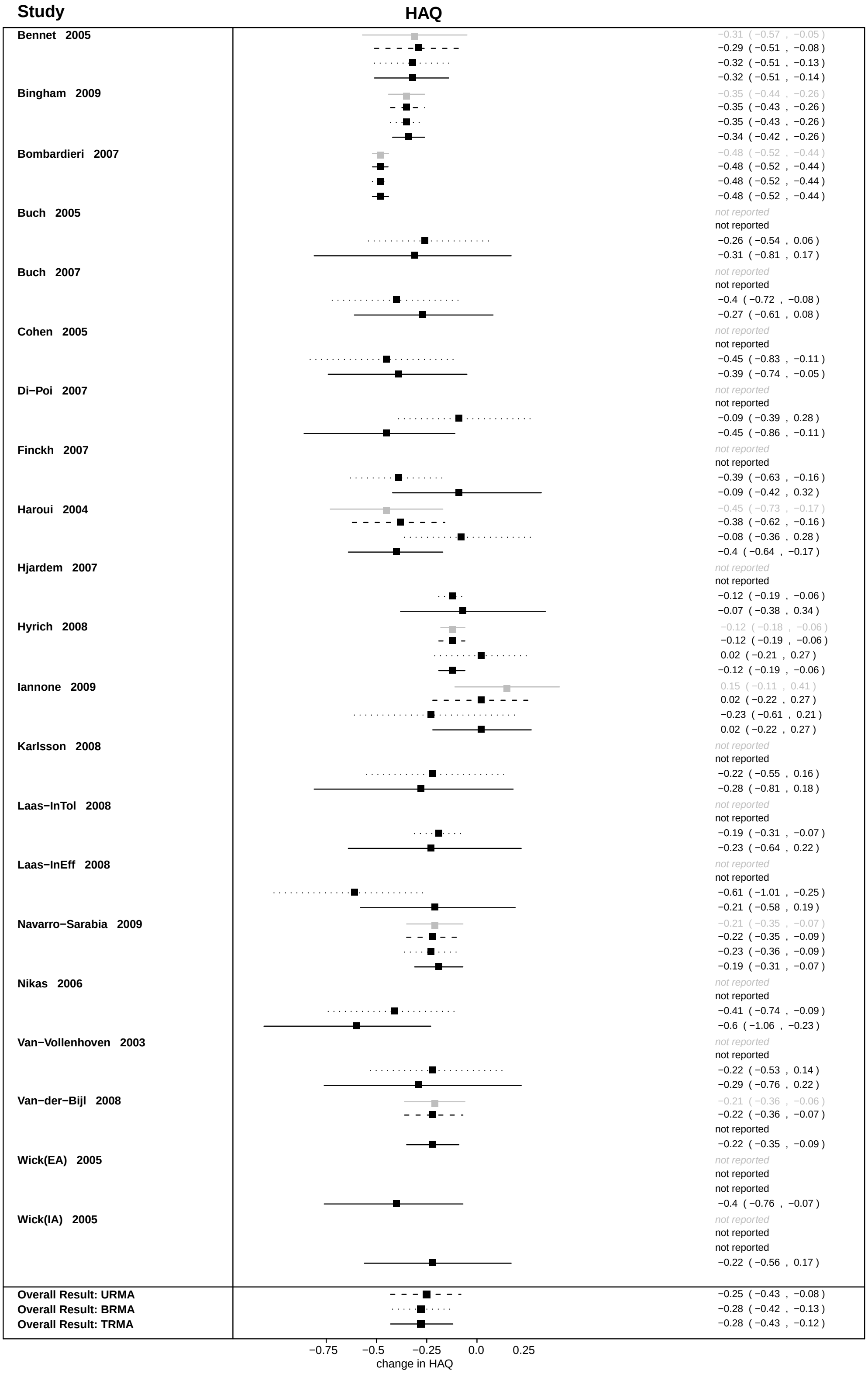

Figure 1. Forest plot for estimates of HAQ from URMA, BRMA and TRMA. Graph shows estimates from the systematic review with 95% CIs (grey solid lines), the "shrunken" and the pooled estimates with 95% CrIs (black lines); URMA: dashed lines, BRMA: dotted lines and TRMA: solid lines.

Copyright © 0000 John Wiley & Sons, Ltd.
